# Weight Teasing and Binge Eating Among Adolescents in Shanghai, China: Role of Anxiety and Unhealthy Weight Control Behaviors

**DOI:** 10.3390/nu17091453

**Published:** 2025-04-26

**Authors:** Landuoduo Du, Yinliang Tan, Wenxin Gu, Zhiping Yu, Zhiruo Zhang, Jingfen Zhu

**Affiliations:** 1 School of Public Health, Shanghai Jiao Tong University, Shanghai 200025, China; melody85@sjtu.edu.cn (L.D.); guwenxin@sjtu.edu.cn (W.G.); 2 Department of Social Medicine and Health Education, School of Public Health, Peking University, Beijing 100191, China; tanyinl@163.com; 3 Department of Nutrition and Dietetics, University of North Florida, Jacksonville, FL 32224, USA; z.yu@unf.edu

**Keywords:** adolescents, weight teasing, anxiety, unhealthy weight control behaviors, binge eating

## Abstract

**Objective:** Weight teasing among adolescents is associated with disordered eating behaviors (DEBs), including unhealthy weight control behaviors (UWCBs) and binge eating (BE). This study aimed to explore the relationships between weight teasing and BE, focusing on the mediating roles of anxiety and UWCBs. **Methods:** Our survey was conducted on 5875 adolescents from Shanghai, consisting of 2974 boys (50.6%) and 2901 girls (49.4%), with an average age of (13.97 ± 2.06) years. A structural equation model was constructed to examine the indirect effects of weight teasing on BE through anxiety and UWCBs, adjusting for gender, age, and their body mass index (BMI). **Results:** Weight teasing demonstrated an indirect association with BE, primarily through UWCBs (β = 0.047, *p* < 0.001) and anxiety (β = 0.038, *p* < 0.001). The chain mediating effect of anxiety and UWCBs was relatively modest (β = 0.017, *p* < 0.001). **Conclusions:** The study highlights the importance of addressing anxiety and weight teasing and promotion of healthy weight control behaviors among adolescents to prevent eating disorders. Future attention to these issues in boys is also warranted.

## 1. Introduction

Disordered eating behaviors (DEBs), which include binge eating (BE) and unhealthy weight control behaviors (UWCBs) [1], are associated with harmful physical and psychological outcomes, even when they do not meet the full criteria for a clinical eating disorder (ED) [2]. It is notable that the prevalence of DEBs in adolescents is much higher than that of full-blown EDs [3]. Without timely and effective interventions, DEBs are likely to progress to the development of a diagnosable ED, characterized by serious psychiatric illnesses involving abnormal eating and/or weight control behaviors [4]. BE, defined as consuming a significantly large amount of food in a brief period time accompanied by a feeling of a loss of control [5], has shown a prevalence ranging from 7.3% to 36.8% among American adolescents [3,6,7]. On the other hand, UWCBs include some unhealthy measures taken to control body weight, such as fasting, taking diet pills and exercising excessively [8]. Despite a 10-year follow-up study indicating a decreasing trend in the prevalence of UWCBs among adolescents in the United States, they have remained relatively high (Male: 39.6%, Female: 58.4% in 1999; Male: 38.1%, Female: 50.2% in 2010) [9]. These statistics emphasize the persistent challenges surrounding adolescent health and the urgent need to address DEBs before they can develop into more severe EDs. Furthermore, DEBs are correlated with heightened susceptibility to a spectrum of health challenges, not only including an elevated risk of obesity, hyperlipidemia, and cardiovascular diseases [10], but also a pronounced vulnerability to severe mental health conditions such as depression, anxiety, and other psychiatric disorders [11].

Given the serious harm of DEBs among adolescents, identifying factors influencing the onset and development of adolescent DEBs is particularly crucial. Studies have indicated that the experience of weight-related teasing in adolescence may lead to an increased occurrence of DEBs [2,12,13]. Weight teasing, a form of body shaming involving criticism or negative comments about an individual’s weight and appearance [14], has been consistently associated with DEBs in previous research [12]. Specifically, studies showed that weight-related teasing has a strong impact on BE [2,15,16]. Chinese adolescents frequently encounter weight-related teasing, particularly if they are overweight. Research conducted in Guangdong province highlighted the elevated risk faced by adolescents with overweight, with the findings indicating that they were 1.60 times more likely to experience bullying for their weight compared to their normal-weight peers (95%CI: 1.18–2.17) [17].

In the Eating and Activity over Time (EAT) 2010–2018 study in the United States, adolescents who had experienced weight teasing were more likely to experience UWCBs (53.5% vs. 35.9%, *p* < 0.001) and BE (12.3% vs. 4.3%, *p* < 0.001) during their young adulthood [2]. Furthermore, weight teasing often led to social exclusion and adverse effects on mental well-being [18]. Individuals subjected to such teasing frequently reported heightened levels of anxiety [15]. This can intertwine with other negative emotions such as stress, potentially creating a fertile ground for the emergence of BE among adolescents [19]. A study conducted in Poland showed that an increase in anxiety scores corresponded to a greater likelihood of engaging in UWCBs (OR = 1.086, *p* = 0.006) [20]. Additionally, a study has revealed a noteworthy correlation between anxiety and different UWCBs, notably fasting, among adolescents [21]. They emphasized the necessity to comprehend the relationship between psychological distress (particularly anxiety) and the adoption of DEBs among adolescents, and to deploy interventions to address them.

**Figure 1:**
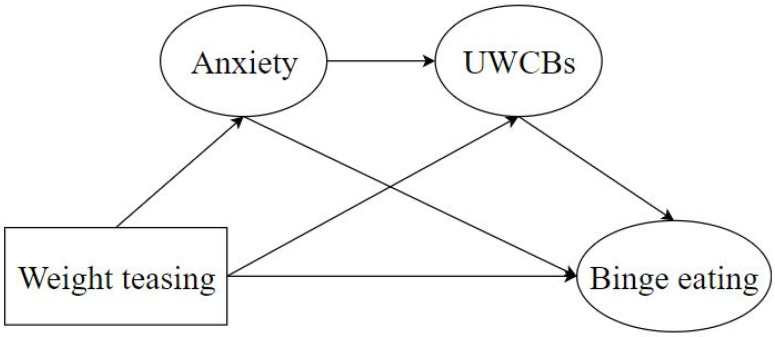
Structural equation model of the relationship between weight teasing and binge eating among adolescents. This model illustrates the relationship between weight teasing and binge eating behavior in adolescents, with a focus on the mediating roles of anxiety and unhealthy weight control behaviors. Note: UWCBs: unhealthy weight control behaviors.

In general, adolescence marks a critical stage in both physical and psychological development, where the emergence of DEBs can exert profound and lasting effects on subsequent life stages. By investigating the factors and mechanisms influencing adolescents’ DEBs, there lies an opportunity to enhance their healthy eating behaviors and reduce their risk of progressing to a clinically diagnosed ED. Drawing from existing research, we hypothesize that weight teasing experienced by adolescents is associated with their binge eating behaviors, and that UWCBs and anxiety may play an important role in this relationship. Our study then employs a structural equation model (SEM) to explore the relationship between weight teasing and BE among adolescents and the roles played by anxiety and UWCBs. The SEM proposed for examination is visually depicted in Figure 1, offering a comprehensive framework to explore the underlying dynamics driving DEBs among adolescents. Through an investigation utilizing SEM, we aim to explore the complex relationships between weight teasing and DEBs, and to provide evidence that may inform future intervention development to support adolescents’ mental health and eating behaviors.

## 2. Materials and Methods

### 2.1. Participants and Procedure

The data for this 2021 cross-sectional study were obtained from a school-based health behaviors cohort among Chinese adolescents in Shanghai, and were collected through multistage and stratified cluster random sampling, with the detailed sampling methods previously described in other articles [22]. Initially, we randomly selected 2 districts from the 16 total districts in Shanghai. Subsequently, 8 schools were randomly selected according to school type, comprising 5 junior high schools (including both private and public schools) and 3 senior high schools (including both key and regular schools). All students enrolled in these selected schools were included in the study, with only a small number excluded due to absence on the day of the survey.

An online questionnaire survey was conducted, resulting in a total of 6070 completed questionnaires. We excluded those who spent insufficient time completing the questionnaire (≤200 s) and those with unreasonable body mass index (BMI) values, for example, a BMI of 7.5 kg/m^2^ (weights of 30 kg with heights of 200 cm). Following these exclusions, a total of 5875 questionnaires were considered valid and subsequently included in the final analysis (resulting in an effective rate of 96.79%). The study was approved by the Ethics Committee of School of Public Health, Shanghai Jiao Tong University School of Medicine (SJUPN202016), and informed consent was obtained from all participating individuals and their guardians prior to their involvement in the study.

### 2.2. Demographic Data

Demographic characteristics included gender (male or female), age (years old), school type (junior high school or senior high school), registered residence (local or nonlocal), and monthly pocket money (<200 RMB, 200–599 RMB, or ≥600 RMB).

### 2.3. Weight Teasing

Questions and classification criteria were developed based on the EAT project [23]. Participants were asked: “*How frequently have you been teased because of your weight?*” with the response options as follows: “never” = 1, “less than once a year” = 2, “several times a year” = 3, “several times a month” = 4, and “at least once a week” = 5. The scores were directly integrated into the structural equation model. Based on previous studies [24,25], participants who reported experiencing weight teasing more than once a year (≥3 points) were considered to have experienced weight teasing, while those who reported less frequent teasing (≤2 points) were considered not to have experienced this.

### 2.4. Anxiety

Anxiety was assessed using The Generalized Anxiety Disorder Scale (GAD-7) [26]. A four-point scale (0 = not at all, 1 = several days, 2 = more than half the days, 3 = nearly every day) was applied to all 7 items. The scores from these questions were integrated into the model. In the descriptive statistics, a total score of less than 5 was indicative of no anxiety symptoms, while a score of 5 or greater was indicative of the presence of anxiety symptoms [26]. The reliability and validity of the questions have been demonstrated in previous studies [27,28]. In the current study, the Cronbach’s α = 0.951.

### 2.5. Unhealthy Weight Control Behaviors

The questionnaire on UWCBs was designed based on the American Youth Risk Behavior Survey (YRBS) [29], and it has been widely adopted in previous studies to assess weight-related behaviors among adolescents [30,31,32]. Participants were asked: “*In the past 4 weeks (28 days), how many times have you used the following behaviors to control your body shape or weight?*” These behaviors included taking diet pills/laxatives/diuretics without a doctor’s advice, vomiting, exercising excessively, eating less food or fewer calories, fasting, and using a food substitute. The use of food substitutes here refers to the consumption of meal replacement powders or a special drink for weight control. Previous Chinese research has also employed specific questions within it to investigate the relationship between adolescent UWCBs, weight status, and health behaviors [33,34]. In the current study, respondents who had engaged in any type of UWCB once or more were considered to have engaged in this kind of UWCB [35].

Given the differences in the study population, this research did not follow the classification of UWCBs used by American scholars [35]. Instead, these six UWCBs were re-categorized into two groups: extreme UWCBs (taking diet pills/laxatives/diuretics, vomiting, exercise excessively, and fasting) and other UWCBs (eating less food or fewer calories and using a food substitute) through factor analysis. Following data standardization, the Kaiser–Meyer–Olkin value was 0.785, and Bartlett’s test was significant (*p* < 0.001), indicating the suitability of the data for factor analysis. Considering both eigenvalues and interpretability, two components were extracted, explaining 62.34% of the total variance. The number of each type of UWCB was incorporated into the model.

### 2.6. Binge Eating

The survey on BE was based on the Questionnaire on Eating and Weight Patterns-Revised [36] and the EAT project [37]. The questionnaire contained two questions (BE1 and BE2), “*In the past 4 weeks (28 days), how many times have you eaten a very large meal (larger than your usual meal)?*” and “*In the past 4 weeks (28 days), how many times have you eaten a very large meal (larger than your usual meal) and felt unable to control yourself at the time?*”. Response options included “never” = 1, “one to four times” = 2, “five to eight times” = 3, and “more than eight times” = 4. Scores from these questions were integrated into the model. Participants who scored two or more points for both questions were considered to have experienced BE [36]. In the current study, the Cronbach’s α = 0.725.

### 2.7. Statistical Analysis

SPSS 24.0 was used to conduct descriptive statistics, factor analysis, and Spearman correlation analyses. A structural equation model (SEM) was used to assess the relationship between weight teasing and binge eating, as well as the role of anxiety and UWCBs through Mplus 7.4. In this model, weight teasing was the independent variable, anxiety and UWCBs were introduced as mediators, and BE was a dependent variable (Figure 1). Gender, age, and BMI were entered as control variables. Model fit was determined by using goodness-of-fit indicators: Root Mean Square Error of Approximation (RMSEA) and Standardized Root Mean Square Residual (SRMR) < 0.05, and Comparative Fit Index (CFI) and Tucker–Lewis Index (TLI) > 0.95. The model provided an acceptable fit to the data (χ^2^/*df* = 12.291; RMSEA = 0.044; SRMR = 0.017; CFI = 0.981; TLI = 0.974). The significance of indirect effects was assessed using the bootstrap procedure (5000 draws), with the level of significance set at 0.05.

## 3. Results

### 3.1. Participant Characteristics

A total of 5875 adolescents were included in the final analysis, comprising 3447 (58.7%) junior high school students and 2428 (41.3%) senior high school students. A total of 64.3% of the participants were local residents, and over half of them had monthly pocket money below 200 RMB. The study group consisted of 2974 boys (50.6%) and 2901 girls (49.4%), with an average age of (13.97 ± 2.06) years. In addition, their average BMI was (20.58 ± 4.38) kg/m^2^.

As shown in Table 1, the prevalence of extreme UWCBs among adolescents was 12.1%, with a higher rate observed in boys compared to girls (*p* < 0.001). Notably, specific extreme UWCBs varied by age: exercising excessively (*p* < 0.001) and fasting (*p* = 0.002) were more prevalent among adolescents aged < 14 years, whereas taking diet pills/laxatives/diuretics (*p* = 0.023) was more common in those aged ≥ 14 years. The prevalence of any type of other UWCBs was 36.0%, with no significant gender difference. Further age-related differences emerged in these behaviors: using a food substitute (*p* < 0.001) was more frequently reported by adolescents aged < 14 years, while eating less food or fewer calories (*p* = 0.001) was more prevalent in those aged ≥ 14 years. Anxiety was prevalent among all participants (34.7%), and the rate of anxiety was higher in girls (38.5%) than in boys (30.9%). Moreover, anxiety was significantly more common in adolescents aged ≥ 14 years (41.6%) compared to their younger counterparts (28.1%). However, the prevalence of BE was higher in boys (32.0%) compared to girls (22.5%). Moreover, 17.1% of the participants had experienced weight teasing, with no gender or age difference.

### 3.2. Correlation Between Weight Teasing and Disordered Eating Behaviors

Correlational analyses revealed significant associations between weight teasing, UWCBs, anxiety, and binge eating, as reported in Table 2.

**Figure 2:**
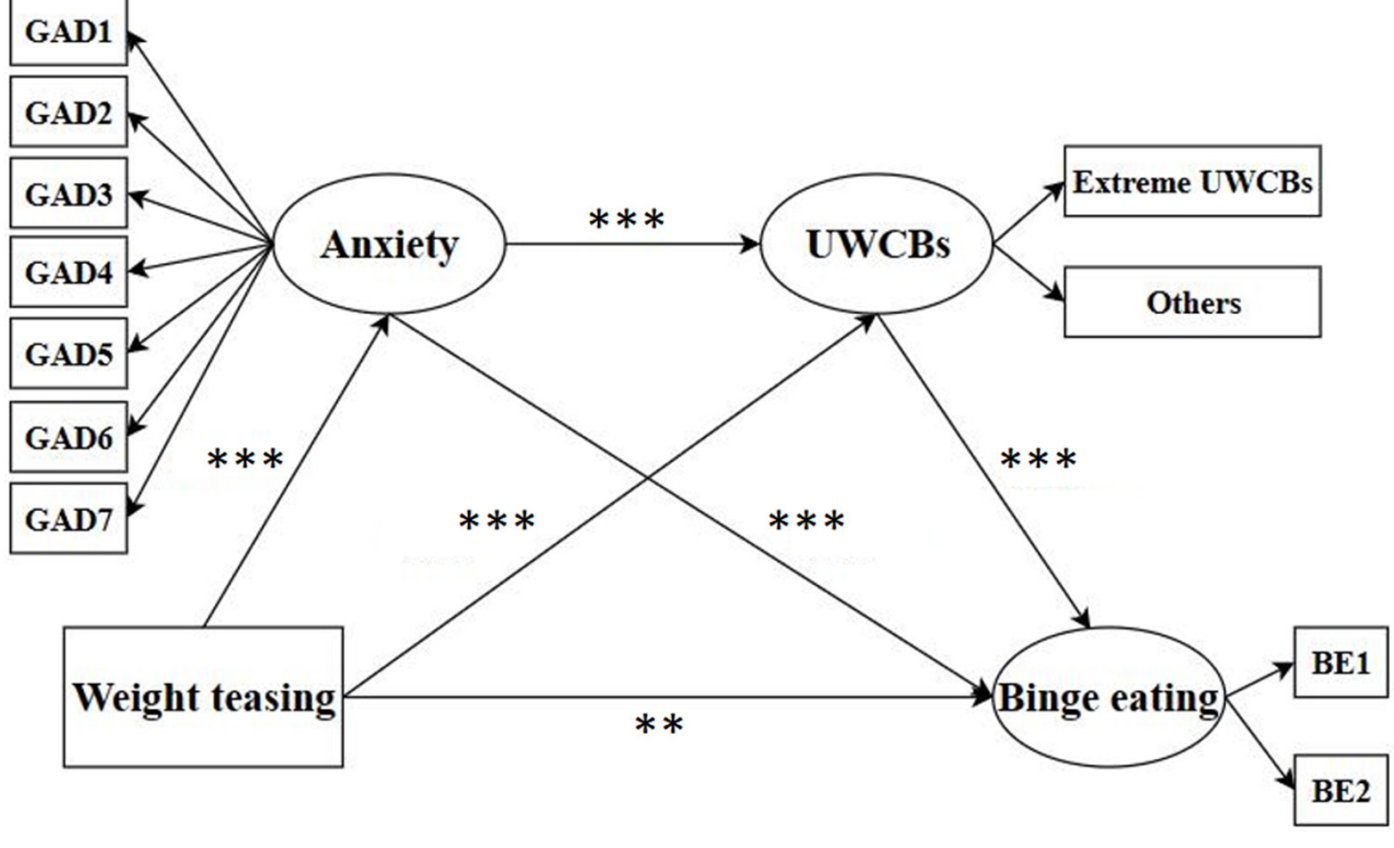
Direct and indirect relationships among weight teasing, unhealthy weight control behaviors, anxiety, and binge eating. The figure highlights the significant mediating effects of UWCBs and anxiety on the relationship between weight teasing and binge eating. Note: Although not shown in the figure, gender, age, and BMI were entered as control variables. *** *p* < 0.001, ** *p* < 0.01. BE: binge eating; UWCBs: unhealthy weight control behaviors.

As shown in Figure 2, all the pathways to BE were statistically significant (*p* < 0.05). As detailed in Table 3, the total effect consisted of a direct effect of 0.061 (95%CI: 0.024–0.096) and a total indirect effect of 0.102 (95%CI: 0.083–0.123), constituting 62.58% of the total effect. Both anxiety and UWCBs emerged as significant mediators. Furthermore, the serial mediating effect of anxiety and UWCBs was also significant (β = 0.017, 95% CI: 0.012–0.023), contributing 10.43% to the total effect.

## 4. Discussion

This study established the relationship between weight teasing and binge eating among adolescents, revealing associations that operate through both direct and indirect pathways. Notably, the indirect effects were particularly prominent, with UWCBs identified as the principal mediators in this relationship. Furthermore, the mediation analysis also uncovered anxiety as a significant factor, acting both independently and through a chain-mediated effect from anxiety to UWCBs. These findings demonstrated some of the complex mechanisms underlying the association between adolescents experiencing weight teasing and BE, and highlighted the role of anxiety and UWCBs.

In this study, it was observed that the severity of BE and extreme UWCBs were notably higher among boys compared to girls. This aligned with the findings from a study conducted in Iran, where there was a higher prevalence of boys engaging in vomiting and using diet pills compared to girls [38]. Studies have indicated that weight teasing or stigmatization experienced by boys can have significant implications on their health, despite such issues historically receiving less attention compared to girls, so that they might have been previously disregarded [39]. This highlighted a critical gap in understanding and addressing the unique challenges faced by boys in relation to their eating and weight control behaviors. By broadening the scope of research and interventions to include the experiences of both genders, we can better support adolescents in cultivating healthy attitudes towards food, weight, and overall well-being. Our findings revealed distinct patterns in UWCBs between adolescents aged < 14 years and those aged ≥ 14 years. The existing research has revealed inconsistent results, and further studies are still needed. For example, a longitudinal study demonstrated that some weight control behaviors, such as vomiting and taking diet pills, increased from early adolescence to adulthood, particularly among females [40]. However, research has identified fasting behaviors emerging as early as middle school and persisting throughout adolescence in females [41]. This suggests that these behaviors may emerge early and remain stable over time. One potential explanation is that adolescents are highly susceptible to media influences, and studies have shown that exposure to social media significantly amplifies body dissatisfaction in adolescents, which may drive them to adopt UWCBs to lose or maintain weight [42,43]. Different age groups may have a varying understanding of the message carried by social media, thus leading to different behavioral choices.

It is important that the present study identified UWCBs as vital mediators between weight teasing and BE in adolescents. Prior research has indicated that weight-based teasing may lead to the occurrence of UWCBs [44,45]. Sociocultural theories have proposed that weight teasing from family members or friends can adversely impact adolescents’ body image, propelling them to strive for an ideal appearance and thus elevating the risk of UWCBs [8]. Within the family context, the protective effect of regular family meals against UWCBs could be compromised if discussions about weight-related issues occur during family meal times [46]. Similarly, outside of the family sphere, peers who engage in unhealthy dietary and weight control behaviors may serve as a source of weight teasing. Adolescents, in their quest for social acceptance, may be inclined to emulate the behaviors of their friends, even at the expense of their own well-being [47], thereby heightening their risk of developing disordered eating patterns. There may be a bidirectional relationship between UWCBs and BE, but further research is still needed to explore this relationship. Research has indicated that female adolescents who pursue an ideal body shape and experience body dissatisfaction might engage in UWCBs, thereby increasing the risk of BE [40]. On the other hand, a longitudinal study found that adolescents’ BE might predict their subsequent UWCBs [48]. This may be because BE can intensify their fear of weight gain and body dissatisfaction, thereby enhancing the expectancy of thinness and leading to the adoption of UWCBs [48,49].

In addition to uncovering the role of UWCBs, this study revealed that anxiety also played a significant role in mediating the complex relationship between weight teasing and BE among adolescents. This finding aligned with a study involving Iranian high school students [50], where psychological disorders such as depression, anxiety, and stress were identified as key mediators of the link between weight stigma and BE. Moreover, Chinese research has provided further support for this notion, suggesting that weight teasing is directly or indirectly associated with adolescents’ dietary disturbances through psychological distress, with peer support playing a regulatory role in this context [51]. The relationship between anxiety and BE may be explained by theories on comfort eating, which posit that individuals turn to DEBs to cope with negative emotions [52], ultimately leading to an increase in appetite. Moreover, our study showed that the sequential effects of anxiety and UWCBs also indirectly mediated the association between weight teasing and BE. This observation was consistent with a study indicating that higher anxiety scores among girls were linked to a higher prevalence of UWCBs (*p* = 0.006) [20]. Adolescents experiencing body dissatisfaction and negative emotions as a result of weight teasing may be driven to alleviate their distress by attempting to lose weight, thereby elevating the risk of DEBs, especially among individuals with overweight [53].

While our study primarily aimed to substantiate the correlation between weight teasing, anxiety, and DEBs, it is essential to acknowledge that the relationship between these variables may not be unidirectional. Indeed, research has suggested that the associations between them can manifest bidirectionally. For instance, while weight teasing and anxiety may contribute to the development of DEBs, it is noteworthy that engagement in UWCBs can also pose a threat to the psychological well-being of adolescents. Research indicated that adopting UWCBs during adolescence, such as vomiting or inappropriately using laxatives in the absence of effective weight loss, increased feelings of shame and frustration [54]. These negative emotions could elevate symptoms of anxiety and depression, creating a vicious cycle of psychological distress. Similarly, scholars have proposed that BE may not only be a consequence of anxiety and depression but can also engender feelings of self-disgust and be accompanied by anxiety and depression [55]. Moreover, studies have also suggested that adolescents with binge eating behaviors exhibit higher levels of social anxiety [56], indicating the bidirectional relationship between DEBs and various manifestations of anxiety.

Our study had several notable strengths. Firstly, it boasted a substantial sample size comprising students across all stages of secondary school, thereby ensuring the comprehensive representation and generality of findings. This allowed for a nuanced understanding of DEBs in adolescents at different developmental stages, enriching the depth of insights gained from the study. Secondly, the present study highlighted the crucial role of UWCBs in the relationship between weight teasing and BE among adolescents, which has received limited attention in previous studies.

Notwithstanding, certain limitations of this study need to be taken into account. First of all, the data were collected solely in Shanghai, China, potentially limiting the generalizability of the research findings to other regions or countries with different cultural contexts and socioeconomic conditions. Secondly, all the data collected in this study were self-reported, which may have introduced the possibility of reporting biases and affected the accuracy of the results. Additionally, being a cross-sectional survey, this study cannot establish causality; hence, further exploration of the causal relationship between various factors is required through longitudinal experimental studies. Finally, the COVID-19 pandemic may have affected the associations between the variables we studied, especially its effects on adolescents’ psychological and emotional well-being. Future research should consider the potential influence of the pandemic on the outcomes of interest.

## 5. Conclusions

This study contributes further evidence to our understanding of the relationship between weight teasing and binge eating (BE) in adolescents. Specifically, unhealthy weight control behaviors and anxiety are identified as mediators in this relationship. These findings emphasize the need for future longitudinal research to explore potential causal pathways. Additionally, the observed gender differences—particularly the elevated risks of BE and extreme unhealthy weight control behaviors among boys—warrant more attention in future research.

## Figures and Tables

**Table 1:** Prevalence of UWCBs ^a^, BE ^b^, weight teasing, and anxiety, and their distributions by gender and age [N (%)].

	Total	Gender		χ²	*p*	Age		χ²	*p*
		Male	Female			<14 Years	≥14 Years		
UWCBs (extreme)									
Any (type)	711 (12.1)	413 (13.9)	298 (10.3)	18.04	<0.001	440 (14.6)	271 (9.5)	35.11	<0.001
Taking diet pills/laxatives/diuretics	132 (2.3)	97 (3.3)	35 (1.2)	28.24	<0.001	55 (1.8)	77 (2.7)	5.20	0.023
Vomiting	194 (3.3)	139 (4.7)	55 (1.9)	35.49	<0.001	95 (3.1)	99 (3.5)	0.50	0.478
Exercising excessively	500 (8.5)	295 (9.9)	205 (7.1)	15.35	<0.001	315 (10.4)	185 (6.5)	29.08	<0.001
Fasting	352 (6.0)	205 (6.9)	147 (5.1)	8.69	0.003	209 (6.9)	143 (5.0)	9.36	0.002
UWCBs (others)									
Any (type)	2114 (36.0)	1041 (35.0)	1073 (37.0)	2.51	0.113	1105 (36.5)	1009 (35.4)	0.84	0.359
Eating less food or fewer calories	1502 (25.6)	746 (25.1)	756 (26.1)	0.74	0.391	717 (23.7)	785 (27.5)	11.28	0.001
Using a food substitute	1172 (20.0)	620 (20.9)	552 (19.0)	3.04	0.081	692 (22.9)	480 (16.8)	33.61	<0.001
BE	1606 (27.3)	953 (32.0)	653 (22.5)	67.21	<0.001	830 (27.4)	776 (27.2)	0.04	0.844
Weight teasing	1002 (17.1)	480 (16.1)	522 (18.0)	3.57	0.059	536 (17.7)	466 (16.3)	1.98	0.160
Anxiety	2036 (34.7)	918 (30.9)	1118 (38.5)	38.16	<0.001	851 (28.1)	1185 (41.6)	116.80	<0.001

^a^ unhealthy weight control behaviors, ^b^ binge eating.

**Table 2:** Correlation coefficients of observed variables.

	Weight Teasing	UWCBs (Extreme)	UWCBs (Others)	GAD	BE1	BE2
Weight teasing	1					
UWCBs ^a^ (extreme)	0.119 ***	1				
UWCBs (others)	0.193 ***	0.287 ***	1			
GAD	0.214 ***	0.163 ***	0.150 ***	1		
BE ^b^ 1	0.097 ***	0.107 ***	0.085 ***	0.123 ***	1	
BE2	0.148 ***	0.149 ***	0.181 ***	0.179 ***	0.506 ***	1

*** *p* < 0.001, ^a^ unhealthy weight control behaviors, ^b^ binge eating.

**Table 3:** Standardized estimates of direct and indirect effects of weight teasing on BE ^a^.

Path	β	95%CI		Relative Effect Value
		LLCI	ULCI	
Direct effects				
Weight teasing→BE ^a^	0.061	0.024	0.096	37.42%
Indirect effects				
Weight teasing→anxiety→BE	0.038	0.026	0.051	23.31%
Weight teasing→UWCBs ^b^→BE	0.047	0.033	0.065	28.83%
Weight teasing→anxiety→UWCBs→BE	0.017	0.012	0.023	10.43%
Total indirect effects	0.102	0.083	0.123	62.58%

LLCI = lower limit in 95% confidence interval. ULCI = upper limit in 95% confidence interval. ^a^ binge eating. ^b^ unhealthy weight control behaviors.

## Data Availability

The original contributions presented in this study are included in the article. Further inquiries can be directed to the corresponding author.

## Abbreviations

The following abbreviations are used in this manuscript:

BE	Binge eating
BMI	Body mass index
DEBs	Disordered eating behaviors
UWCBs	Unhealthy weight control behaviors
